# When enough is enough: how the decision was made to stop the FEAST trial: data and safety monitoring in an African trial of Fluid Expansion As Supportive Therapy (FEAST) for critically ill children

**DOI:** 10.1186/1745-6215-14-85

**Published:** 2013-03-26

**Authors:** Jim Todd, Robert S Heyderman, Philippa Musoke, Tim Peto

**Affiliations:** 1 London School of Hygiene and Tropical Medicine, Keppel Street, London WC1E 7HT, UK; 2 Kilimanjaro Christian Medical University College, PO Box 2240, Moshi, Tanzania; 3 Malawi Liverpool Wellcome Trust Clinical Research Program, University of Malawi College of Medicine, Blantyre, Malawi; 4 Department of Pediatrics, Makerere University School of Medicine, PO Box 7072, Kampala, Uganda; 5 Nuffield Department of Medicine, John Radcliffe Hospital, Headley Way, Headington, Oxford, OX3 9DU, UK; 6 Department of Epidemiology and Biostatistics, KCMC, PO Box 2240, Moshi, Tanzania

## Abstract

In resource-rich countries, bolus fluid expansion is routinely used for the treatment of poor perfusion and shock, but is less commonly used in many African settings. Controversial results from the recently completed FEAST (Fluid Expansion As Supportive Therapy) trial in African children have raised questions about the use of intravenous bolus fluid for the treatment of shock. Prior to the start of the trial, the Independent data monitoring committee (IDMC) developed stopping rules for the proof of benefit that bolus fluid resuscitation would bring. Although careful safety monitoring was put in place, there was less expectation that bolus fluid expansion would be harmful and differential stopping rules for harm were not formulated.

In July 2010, two protocol amendments were agreed to increase the sample size from 2,880 to 3,600 children, and to increase bolus fluid administration. There was a non-significant trend against bolus treatment, but although the implications were discussed, the IDMC did not comment on the results, or on the amendments, in order to avoid inadvertent partial unblinding of the study.

In January 2011, the trial was stopped for futility, as the combined intervention arms had significantly higher mortality (relative risk 1.46, 95% CI 1.13 to 1.90, *P* = 0.004) than the control arm. The stopping rule for proof of benefit was not achieved, and the IDMC stopped the trial with a lower level of significance (*P* = 0.01) due to futility and an increased risk of mortality from bolus fluid expansion in children enrolled in the trial. The basis for this decision was that the local standard of care was not to use bolus fluid for the care of children with shock in these African countries, and this was a different standard of care to that used in the UK. These decisions emphasize two important principles: firstly, the IDMC should avoid inadvertent unblinding of the trial by commenting on amendments, and secondly, when considering stopping a trial, the IDMC should be guided by the local standard of care rather than standards of care in other parts of the world.

## Background

The FEAST (Fluid Expansion As Supportive Therapy) trial was a multicenter randomized controlled trial of rapid fluid resuscitation in African children with features of shock [1]. Fluid expansion is routine emergency therapy in the management of shock in resource-rich countries [2], however, the use of fluid resuscitation in severely ill children is less common in sub-Saharan Africa. Moreover, the international evidence for fluid expansion is largely subjective, and the most recent review graded the pediatric recommendation (20mls/kg boluses over 5 to 10 minutes up to 60mls/kg) as 2C, indicating a weak recommendation with low quality of evidence [3]. Prior to the start of the FEAST trial, ethical concerns expressed by some experts about the control arm were raised as children in the control arm would only receive maintenance fluids, in line with the current standard of care in Kenya, Tanzania and Uganda. The trial was stopped early by the Independent data and safety monitoring committee (IDMC) because of lack of benefit and potential harm from bolus fluid compared to children in the control arm.

IDMCs are required to ensure the safety of trial participants and guarantee integrity of the trial data [4] but the deliberations of the IDMC are not often published. Previous papers have reported on the conduct of the IDMCs after study results have been published [5,6]. The FEAST trial results were surprising, since they question decades of practice in resource-rich countries and have produced controversy in subsequent commentaries, discussion forums and correspondence [7-11]. Two questions have arisen: firstly, should the IDMC have approved a protocol amendment increasing the bolus dose [7]; and secondly, was the trial stopped at the right time? This paper highlights the challenges faced in making these key decisions.

### The FEAST trial

There were two separate studies within the FEAST trial (FEAST A and B). FEAST B was restricted to children with severe febrile illness complicated by severe hypotension (defined as a systolic blood pressure < 50 mmHg if < 12 months old; < 60 mmHg if 1 to 5 years old; < 70 mmHg if > 5 years old) who were randomized to albumin or saline boluses of 40 to 60mls/kg only. The numbers enrolled were too small (N = 29) to be assessed and FEAST B will not be considered further. In FEAST A, children had severe febrile illness and impaired perfusion but without severe hypotension. Children were randomized to one of three arms: saline bolus 20 ml/kg (increased to 40 ml/kg after protocol amendment), albumin bolus 20 ml/kg (increased to 40 ml/kg after protocol amendment), or control (no bolus: maintenance fluids 4 ml/kg/hour), with the two primary comparisons between saline and control arms (to address whether fluid boluses resulted in a better outcome than standard of care), and between albumin and saline arms (examining whether a colloid resuscitation fluid (5% human albumin solution) was superior to an inexpensive crystalloid solution (0.9% saline)). In addition, children in all three arms received the current standard of care, which included intravenous antibiotics, antimalarial drugs (for those with *Plasmodium falciparum* malaria) and intravenous maintenance fluids (between 2.5 and 4mls/kg/hour as per national guidelines) until the child was able to retain oral fluids. Antipyretics, anticonvulsants and treatment for hypoglycemia (blood sugar < 2.5mmols/L) were administered according to nationally agreed protocols. Children with a hemoglobin < 5 g/dl were transfused with 20mls/kg of whole blood over four hours. Children were managed on general pediatric wards; mechanical ventilation (other than short-term ‘bag-and-mask’ support) was not available. Basic infrastructural support for emergency care, oxygen saturation and automatic blood pressure monitors were provided for each site. Training in triage and emergency pediatric life support was given prior to and throughout the trial to optimize case recognition, implement supportive management and ensure protocol adherence.

**Figure 1:**
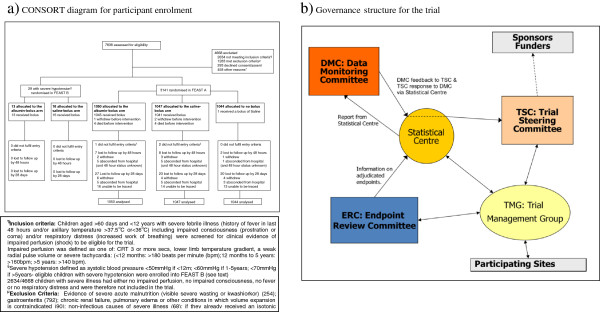
**Overview of FEAST trial. (a) **CONSORT diagram for participant enrolment, **(b) **Governance structure for the trial.

The primary endpoint for the trial was mortality, however, the IDMC suggested to the trial steering committee that it would also be important to monitor secondary outcomes, which in view of the potential for fluid overload, included severe adverse events (SAE) and some pre-specified adverse events (AE) associated with fluid bolus administration. The trial recorded SAE, and actively solicited the selected AE, and these were reviewed within two days by an independent clinician who removed information about fluid management. An independent Endpoint review committee (ERC), blinded to the randomization arms, reviewed all SAEs and solicited AEs and adjudicated on the event and its association with the intervention. This information was included in the reports to the IDMC. The Trials steering committee (TSC) was responsible for overall governance of the trial (Figure 1b); while the Trial management group (TMG) had day-to-day responsibility for the trial.

### Structure and role of the FEAST IDMC

The IDMC monitored unblinded interim data and, after each meeting, recommended action to the TSC (Table 1). At each meeting, the trial statistics center produced an open report with information pooled across treatment arms (also circulated to the TMG) and a ‘closed report’, only available to the IDMC, detailing protocol implementation and both primary and secondary outcome measures by treatment arm. IDMC meetings had three sessions: an administrative session for IDMC members only, an open session and a final closed session. In open sessions, TMG representatives reported on trial progress and relevant changes in local clinical practice.

**Table 1:** Possible recommendations from the FEAST charter for the IDMC included


∙	No action needed, trial continues
**∙**	Early stopping due to clear benefit, or harm of a treatment
**∙**	Stopping recruitment within a pre-defined subgroup
**∙**	Stopping one or more arms of the trial and recommendation to amend trial
**∙**	Proposing or commenting on proposed protocol changes
**∙**	Commenting on the Statistical Analysis Plan

The FEAST trial was designed to provide evidence that inform policy makers about the benefits of using a fluid bolus in children admitted to hospital with severe febrile illness. The IDMC charter did not specify explicit stopping rules for the trial, but the protocol required ‘proof beyond reasonable doubt’ that one treatment arm was better than its comparator [12,13]. In the first meetings of the IDMC, the committee discussed the protocol requirements and agreed that the trial should be stopped if proof of benefit for the fluid bolus arms achieved the Haybittle-Peto criteria with a *P*-value < 0.002. However, although the harm from fluid overload in children receiving the bolus was discussed, a different stopping rule for proof of harm was not formulated. The adoption of a single *P*-value implied a symmetric two-sided stopping rule that the trial would be stopped if the results unambiguously identified best clinical practice. In later discussions, the IDMC agreed that an asymmetric stopping rule should be adopted with a lower threshold for stopping the trial if it was found that the new treatment (fluid bolus) was not as good as the local standard of care given in the control arm. However, there was no explicit decision on the *P*-value that would be used for stopping the trial if proof of harm was found.

### The monitoring process

Five interim reports on trial conduct, efficacy, and safety outcomes were prepared for IDMC meetings between June 2009 and January 2011. The main activities, requests and recommendations from the first three IDMC meetings are summarized in Table 2. Data from the third interim analysis included a total of 139 deaths and 30 non-fatal SAEs (Tables 3 and 4).

**Table 2:** IDMC recommendations from first four meetings


**First Meeting (25 February 2009):**	
**∙**	Requested details of adverse event reporting and listings of all individual events by patient identification
**Second Meeting (18 June 2009):**	
**∙**	Requested information on children screened for the trial but not enrolled
**∙**	Requested the number and quantity of additional fluids (other than boluses) given to all children
**∙**	Asked the study teams to make extra effort to ensure that all children attend for neurological examination at 28 days
**Third meeting (12 October 2009):**	
**∙**	Endorsed the review procedure for SAE proposed, and stressed the importance of blinded review by the ERC
**∙**	Accepted co-enrollment of children into other studies
**∙**	Noted planned sub-studies
**Fourth meeting (26 January 2010, third interim analysis):**	
**∙**	Observed improvement in attendance for neurological follow up at 28 days
**∙**	Referred the request by a local IRB for representation on the IDMC to the TSC
**∙**	Requested more detailed information on all fluids received in each arm by time from randomization
**∙**	Agreed with the proposed plan of analysis

**Table 3:** Mortality in FEAST A presented in each IDMC report

					**Comparisons between arms for mortality at 48 hours**		
	**Mortality in each arm**				**Primary endpoint**		**Secondary endpoint**
**Meeting**	**Albumin**	**Saline**	**Control**	**Total**	**Risk ratio for saline bolus versus no bolus**	**Risk ratio for albumin bolus versus saline bolus**	**Risk ratio bolus versus no bolus**
18 June 2009 (data to 13 May 2009); N = 406	12/137 (8.8%)	14/136 (10.3%)	18/133 (13.5%)	44/406 (10.8%)	0.76 (0.39 to 1.47);*P *= 0.41	0.87 (0.42 to1.81); *P *= 0.71	0.70 (0.40 to 1.24); *P *= 0.22
12 October 2009 (data to 31 August 2009); N = 844	30/283 (10.6%)	28/282 (9.9%)	31/279 (11.1%)	89/844 (10.5%)	0.89 (0.06 to 1.45); *P *= 0.65	1.07 (0.66 to 1.74); *P *= 0.79	0.92 (0.61 to 1.39); *P *= 0.71
26 January 2010 (data to 31 December 2009); N = 1,337	50/444 (11.3%)	46/445 (10.3%)	43/448 (9.6%)	139/1337 (10.4%)	1.07 (0.73 to 1.60); *P *= 0.71	1.09 (0.75 to 1.59); *P *= 0.66	1.13 (0.78 to 1.58); *P *= 0.50
26 July 2010 (data to 15 June 2010); N = 2,196	74/732 (10.1%)	74/733 (10.1%)	56/731 (7.7%)	204/2196 (9.3%)	1.32 (0.95 to 1.84); *P *= 0.10	1.001 (0.74 to 1.36); *P *= 0.99	1.32 (0.98 to 1.77); *P *= 0.06
12 January 2011 (data to 15 November 2010); N = 2,987	101/989 (10.2%)	101/989 (10.2%)	69/989 (7.0%)	271/2987 (9.1%)	1.46 (1.09 to 1.96);*P *= 0.01	1.00 (0.77 to 1.30); *P *= 1.0	1.46 (1.13 to 1.90);*P *= 0.004

**Table 4:** Non-fatal SAEs and neurological sequelae by fluid arm

	**Non-fatal SAEs and neurological sequelae^a^ in each arm**			
**Meeting**	**Albumin**	**Saline**	**Control**	**Total**
18 June 2009 (data to 13 May 2009); N = 406	NFSAE: 2	NFSAE: 1	NFSAE: 2	NFSAE: 5
	RICP: 1	other: 1	AR: 1	RICP: 1
	AR: 1		other: 1	AR: 2
				other:2
	NS: 5	NS: 4	NS: 3	NS : 11
12 October 2009 (data to 31 August 2009); N = 844	NFSAE: 3	NFSAE: 2	NFSAE: 2	NFSAE: 7
	RICP: 1	AR: 1	AR: 2	RICP: 1
	AR: 1	other: 1		AR: 3
	other: 1			other: 2
	NS: 9	NS: 12	NS: 5	NS: 16
26 January 2010 (data to 31 December 2009); N = 1,337	NFSAE: 3	NFSAE: 4	NFSAE: 3	NFSAE: 10
	RICP: 1	AR:2	AR: 2	RICP: 1
	AR: 1	other: 2	other: 1	AR: 5
	other: 1			other: 4
	NS: 14	NS: 17	NS: 9	NS: 30
26 July 2010 (data to 15 June 2010); N = 2,196	NFSAE: 4	NFSAE: 8	NFSAE: 4	NFSAE: 16
	RICP: 1	RICP: 1	AR: 3	RICP: 2
	AR: 2	AR: 4	other: 1	AR: 9
	other: 1	other: 3		other: 5
	NS: 13^b^	NS: 10^b^	NS: 7^b^	NS: 20^b^
12 January 2011 (data to 15 November 2010); N = 2,987	NFSAE: 6	NFSAE: 8	NFSAE: 5	NFSAE: 19
	PE: 1	RICP: 1	RICP: 1	PE: 1
	- RICP: 1	- AR: 4	- AR: 3	- RICP: 3
	AR: 3	other: 3	other:1	AR: 10
	other: 1			other: 5
	NS: 22	NS: 16	NS: 10	NS: 38

NFSAE: non-fatal serious adverse event, RICP = raised intracranial pressure, PE = pulmonary edema, AR = allergic reaction, NS = neurological sequelae.

^a^either reviewed and accepted by the ERC or not reviewed at the time of the report.

^b^the definition of neurological sequelae was refined between the reports in 2010 to take into account information about whether each ‘abnormal neurological finding’ at 28 days was an existing condition at baseline. Thus, children with a known neurological abnormality at baseline that had not deteriorated during follow-up were not included in the neurological sequelae numbers from that point onwards.

### Protocol amendments

Following the fourth report, the TMG informed the IDMC of two proposed changes to the trial protocol. Data to June 2010 showed the global mortality was 9.3%, lower than the 11% assumed in the initial sample size calculations, so an increase in the sample size from 2,880 to 3,600 was proposed so that 350 deaths across the three trial arms would be observed. The IDMC noted the TMG request to increase the sample size, and agreed with the rationale for the increase, but did not comment on the increased sample size. The unblinded results showed that the differences between the arms was smaller than expected, and although not statistically significant, in the wrong direction. However, these results were not made available to the TSC and were not used to support the proposed increase in the sample size.

The second proposed change was to increase the fluid volume in both bolus arms during the first hour from 20 ml/kg to 40 ml/kg, which was the usual recommendation for management of children with shock in high-income countries [14]. In the original design of the trial, owing to safety concerns about the possibility of pulmonary edema in children receiving a large initial fluid bolus, the initial protocol specified that children receive 20 ml/kg bolus in the first hour, followed by a further 20 ml/kg bolus in the second hour if shock was ongoing and there were no safety concerns. However, the open report indicated that only 16% of eligible children received a second bolus, only two children had non-fatal, fluid-specific SAEs, both with evidence of raised intracranial pressure (Table 4), and that only 22 children with severe hypotensive shock had been with enrolled in FEAST B. The TSC had three reasons for recommending an increase. Firstly, the bolus fluid volume (20 ml/kg) (rather than 20 ml/kg plus 20 ml/kg as anticipated in the trial design) may be insufficient to answer the study question (that is futility). Secondly, by not following international guidelines on fluid bolus volume, the results would not convince policymakers on the validity of findings. Finally, only 1% of trial participants had severe hypotensive shock (versus 5% predicted in the trial design), which may affect the saline versus albumin comparison (secondary endpoint).

**Figure 2:**
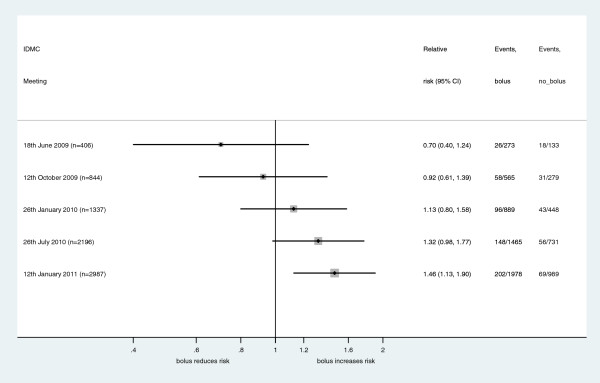
Forest plot of the secondary endpoint of bolus compared to no bolus at IDMC meetings during the FEAST trial.

The IDMC noted 204 deaths (74 albumin bolus, 74 saline bolus, 56 control) from 2,196 children with (Table 2). The relative risk for saline bolus relative to control was 1.32 (95% confidence interval (CI): 0.95 to 1.84, *P* = 0.10). The relative risk for albumin bolus relative to saline bolus was 1.00 (95% CI: 0.74 to 1.36, *P* = 0.99). The combined bolus arms had a small, but not statistically significant, increase in absolute risk of 48-hour mortality of 2.4% (95% CI: -0.2% to 0.5% *P* = 0.063) compared to the control arm (Figure 2). There were 71 non-fatal SAEs (25 albumin bolus, 27 saline bolus, 19 control), and showed no instances of pulmonary edema in any arm. Data on the timing of all fluids given within the trial showed the mean fluid volume in the first hour was 21.4mls/kg and 1.5mls/kg in bolus arms and control arm respectively, and in the second hour was 7.2 mls/kg and 2.9 mls/kg in the bolus arms and control arm respectively, indicating excellent protocol adherence. As the increased fluid bolus was within international guidelines for fluid administration in these children, the IDMC did not ask for further analysis of the interim data as this would not have produced a definitive result and may have prematurely unblinded study investigators to the accumulating results.

The IDMC, satisfied that the protocol was being followed correctly, and that the data did not provide a conclusive result, recommended the trial continue. The report from the IDMC to the TSC had ‘no concerns about safety in any of the three arms’, and ‘noted the change in sample size, and in the fluid management proposed’ and had ‘no issues with the amendments’, but was careful to avoid unblinding of the study by consideration of unblinded results.

### The fifth and final interim report (12 January 2011)

The IDMC reviewed data on 2,987 children. 48-hour mortality was 10.2% in albumin bolus, 10.2% saline bolus and 7.0% in control arms. The relative risk for saline bolus compared to control was 1.46 (95% CI: 1.09 to 1.96, *P* = 0.01) with no evidence of any difference between saline bolus and albumin bolus - relative risk 1.00 (95% CI: 0.74 to 1.36). Comparing the combined intervention arms against control (a secondary comparison) gave a highly significant result (*P* = 0.004), although it had not crossed the implicit threshold (*P* = 0.002) for proven benefit that was set at the beginning of the study. The IDMC discussed and agreed that a stopping rule for proven harm would require less evidence, and the results were adequate to show the futility of continuing with the bolus fluid intervention. The IDMC unanimously recommended immediate cessation of the study recruitment as there was no prospect that the intervention arms could be superior to the control arm (standard of care in Africa), despite knowing that the intervention arms were standard of care in high income countries.

The IDMC recommendation, but not the result of the trial, was communicated to the chief investigator and the TSC chair, who informed site leaders, and immediately stopped the trial. Confidentiality of the results (including primary outcome) was maintained so that the implications could be discussed at a TSC meeting convened on 19 January 2011 in Nairobi, Kenya before locking the database and unblinding. The manuscript was submitted for publication on 6 April 2011, and published on 26 May 2011 [1].

### Results seen by the IDMC

The efficacy and safety results seen at IDMC meetings are summarized in Table 3, Table 4 and Figure 2. The 48-hour mortality fell from 13.5% to 7.0% in the control arm, but remained around 10% in the two intervention arms throughout the study.

## Discussion

An IDMC is an important safeguard for individuals participating in studies. Clinical trials with mortality or serious morbidity as their main outcome need a properly constituted IDMC to independently monitor the conduct of the trial and examine data on safety and efficacy in order to protect the integrity of the trial and the safety of the participants. The terms and conditions of the IDMC should be defined beforehand and should include guidance on the recommendations open to the IDMC and the criteria for making these recommendations. However, IDMC members need to use external evidence, and their expert judgment, in deciding whether or not to recommend changing the trial protocol or stopping the trial. In this trial the IDMC made recommendations to the TSC about the conduct of the study, including the issues on the data, the end point review, and the continuation of the trial.

Before the trial started, views on the use of bolus fluid for children admitted to hospital with signs of shock were polarized. Whereas in many high income countries the use of fluid bolus was routine and standard [2], the practice was not widespread in African hospitals [15]. It was unclear whether the trial should be considered as a new intervention in hospitals where rapid fluid resuscitation was not generally used, or if this was the extension of a ‘proven’ intervention into a group of children with different characteristics. The trial had a null hypothesis of no effect and a two-sided alternative hypothesis, so the IDMC applied the same criteria to all three arms. This point was made to institutional review boards who were initially concerned about the ethics of including a control arm in the study. However, in this African setting, the standard of care was largely no bolus fluid, and the stopping rule for proof of benefit for the new intervention was set at 0.002 to ensure that the final significance would not be adversely affected by the interim analyses. Initially, although the potential for harm was discussed, there were no discussions about having a different stopping rule for proof of harm, and this was agreed later by the IDMC, when safety considerations for children receiving the intervention became apparent.

In July 2010, the IDMC was informed about protocol amendments to increase the sample size and to increase the bolus fluid volume. There was higher mortality in both intervention arms compared to the control arm, but there was no indication to stop the trial (Figure 2), and no ‘proof beyond reasonable doubt’ that one treatment arm was better than the others. Although the IDMC was careful not to comment on the unblinded data, some of the blinded investigators considered the IDMC approval as evidence in favor of the bolus treatment. Following the publication of the trial results, concerns were raised about allowing the amendment to increase the initial fluid bolus when interim results suggested the intervention may be harming children in those arms of the trial [7]. The IDMC considered the data without revealing the results, and noted the proposed amendment without conveying approval for the increased initial bolus dose. The IDMC concluded that the benefits of continuing the trial, to reach a statistically significant result, were more important than stopping the trial when there was no definitive proof-of-harm to children receiving the new interventions.

In January 2011, the trial was stopped early with significantly higher mortality (*P* = 0.01) in children in both bolus arms compared to the control arm (combined endpoints *P* = 0.004) although primary comparisons did not cross the boundary for early stopping according to the guidelines in the protocol. The trial was not stopped because of proven superiority of the control versus intervention arms, as this would have required ‘evidence beyond reasonable doubt’. The IDMC stopped the trial for futility, because the standard of care in Africa was the control arm, and there was no prospect that the intervention arms could ever be superior to the control arm. Furthermore, there was good evidence that children may be harmed by continuation of the trial. The IDMC realized that they were applying asymmetric stopping rules but considered this appropriate for the local standard of care. The IDMC may have continued the trial if the standard of care had been bolus fluid administration as stronger evidence is required to establish ‘beyond reasonable doubt’, that a standard of care is inferior, as opposed to showing that a new treatment is inferior.

## Conclusion

The FEAST trial emphasized two important principles that underpin the role of the IDMC in the conduct of trials. Firstly, the IDMC should not comment on protocol amendments that might lead to inadvertent partial unblinding of the trial and was correct in noting the protocol amendment increasing the bolus dose without conveying information about the results of the trial. Secondly, early termination of the trial should be considered when there is good evidence that continuation would put participants at unnecessary risk and there is no realistic possibility that the new treatment could be superior to current standard of care. Additionally, the implications of the results from the FEAST trial may differ between countries and IDMC applied local standards of care to stop the study.

## Abbreviations

AR: allergic reaction; ERC: endpoint review committee; FEAST: Fluid Expansion As Supportive Therapy; IDMC: independent data monitoring committee; IRB: institutional review board; NFSAE: non-fatal serious adverse event; NS: neurological sequelae; PE: pulmonary edema; RICP: raised intracranial pressure; SAE: serious adverse event; TMG: trial management group; TSC: trial steering committee.

## Competing interests

None of the authors have any competing interests to declare.

## Authors’ contributions

JT served on the independent data monitoring committee of the FEAST trial, and wrote the first draft of the manuscript. RH served on the independent data monitoring committee of the FEAST trial and contributed to the writing of the manuscript. PM served on the independent data monitoring committee of the FEAST trial and contributed to the writing of the manuscript. TP was the chair of the independent data monitoring committee of the FEAST trial, contributed to the writing of the manuscript and is the guarantor of the manuscript. All authors read and approved the final manuscript.
